# Secretome of Aggregated Embryonic Stem Cell-Derived Mesenchymal Stem Cell Modulates the Release of Inflammatory Factors in Lipopolysaccharide-Induced Peripheral Blood Mononuclear Cells

**DOI:** 10.22034/ibj.22.4.237

**Published:** 2018-07

**Authors:** Nastaran Mohammadi Ghahhari, Faezeh Maghsood, Saeed Jahandideh, Majid Lotfinia, Shirin Lak, Behrooz Johari, Asaad Azarnezhad, Mehdi Kadivar

**Affiliations:** 1 Department of Biochemistry, Pasteur Institute of Iran, Tehran, Iran; 2 Cellular and Molecular Research Center, Kurdistan University of Medical Sciences, Sanandaj, Iran

**Keywords:** Cell aggregation, Embryonic stem cells, Inflammation, Mesenchymal stem cells, Mononuclear leukocyte

## Abstract

**Background:**

Bone marrow mesenchymal stem cells (BM-MSCs) have emerged as a potential therapy for various inflammatory diseases. Because of some limitations, several recent studies have suggested the use of embryonic stem cell-derived MSCs (ESC-MSCs) as an alternative for BM-MSCs. Some of the therapeutic effects of the ESC-MSCs are related to the secretion of a broad array of cytokines and growth factors, known as secretome. Harnessing this secretome for therapeutic applications requires the optimization of production of secretary molecules. It has been shown that aggregation of MSCs into 3D spheroids, as a preconditioning strategy, can enhance immunomodulatory potential of such cells. In this study, we investigated the effect of secretome derived from human ESC-MSCs (hESC-MSCs) spheroids on secretion of IL-1β, IL-10, and tumor necrosis factor α (TNF-α) from lipopolysaccharide (LPS)-induced peripheral blood mononuclear cells (PBMCs).

**Methods:**

In the present study, after immunophenotyping and considering mesodermal differentiation of hESC-MSCs, the cells were non-adherently grown to prepare 3D aggregates, and then conditioned medium or secretome was extracted from the cultures. Afterwards, the anti-inflammatory effects of the secretome were assessed in an *in vitro* model of inflammation.

**Results:**

Results from this study showed that aggregate-prepared secretome from hESC-MSCs was able to significantly decrease the secretion of TNF-α (301.7 ± 5.906, *p* < 0.0001) and IL-1β (485.2 ± 48.38, *p* < 0.001) from LPS-induced PBMCs as the indicators of inflammation, in comparison with adherent culture-prepared secretome (TNF-α: 166.6 ± 8.04, IL-1β: 125.2 ± 2.73).

**Conclusion:**

Our study indicated that cell aggregation can be an appropriate strategy to increase immunomodulatory characteristics of hESC-MSCs.

## INTRODUCTION

Mesenchymal stem cells (MSCs) are multi-potent stromal cells that are able to adhere to plastic vessels, express specific cell surface antigens and differentiate into mesodermal lineages[1]. MSCs have emerged as a potential therapy for various inflammatory diseases such as graft versus host disease, multiple sclerosis, arthritis rheumatoid, and Crohn’s disease[2]. MSCs, because of their immunomodulatory and trophic properties when transplanted into animal models, can suppress tissue destruction and modulate inflammatory and immune reactions. MSCs have previously been derived from adult tissues such as bone marrow, adipose tissue, dental pulp, and fetal sources (e.g. umbilical cord and amniotic fluid)[3]. However, tissue-derived MSCs possess some limitations. Harvesting MSCs from any of these sources requires invasive procedures as well as the availability of a suitable donor. In addition, after their isolation from different tissues, MSCs can be expanded definitely during *in vitro* culturing and can undergo cellular senescence[4].

More recently, MSCs have been derived from human pluripotent stem cells (PSCs) including embryonic stem cells (ESCs) and induced PSCs; in addition, their therapeutic potential has been demonstrated for various experimental models such as experimental autoimmune encephalomyelitis[5], myocardial infarction[6], liver failure[7], kidney failure[8], inflammatory bowel disease[9], and arthritis[10]. PSC-derived MSCs do not require invasive harvesting, and it is believed that these cells are capable to maintain some of the unique properties of PSCs, such as high proliferative capabilities while possessing advantageous properties of MSCs, such as the inability to form teratoma.

A broad array of cytokines and growth factors produced by MSCs has a considerable potential to treat various inflammatory diseases[11]. These secreted bioactive molecules, referred to as secretome, possess trophic and immunomodulatory activities. Furthermore, several studies have demonstrated that the administration of conditioned medium (CM) from human MSCs including these secretory molecules can improve the experimental models of ischemic heart[12], myocardial infarction[13], multiple sclerosis[14], lung injury[15], acute kidney failure[16], stroke[17], and liver failure[18]. Therefore, harnessing the MSC secretory molecules can open a new window for the treatment of inflammatory and degenerative diseases.

Recently, it has been shown that appropriate manipulation of MSCs in culture might enhance their therapeutic benefits by increased secretion of several soluble cytokines and growth factors that tune the response to injury and/or inflammation. Different strategies have been used to precondition the MSCs before secretome preparation. Some of these strategies include hypoxic exposure, thermal shock induction, pharmacological treatment, and biophysical preconditioning[19]. A study has previously revealed that intravenous-administrated bone marrow MSCs (BM-MSCs) can improve cardiac function and diminish scarring in a mouse model of myocardial infarction. This improvement was because of the trapped MSCs in the lung as microemboli that secreted some anti-inflammatory proteins. More considerations indicated that aggregation of human MSCs into 3D spheroids enhances immunomodulatory and regenerative capacity of these cells[20]. However, the effect of aggregation on immunomodulatory properties of human stem cell-derived MSCs (hESC-MSCs) was not studied until now. In the present study, we first characterized hESC-MSCs and then a non-adherent culture method was used to prepare 3D aggregates. Afterwards, the secretome was extracted from hESC-MSC spheroids, and its biological effects were assessed *in vitro* and compared with adherent hESC-MSCs secretome/2D cultured hESC-MSCs secretome.

## MATERIALS AND METHODS

### Sources of reagents

Human embryonic-derived MSCs (RH6-MSCs) were purchased from the Royan Institute (Iran). All reagents used for cell culture, including Dulbecco’s Modified Eagle’s Medium (DMEM, low glucose), fetal bovine serum (FBS), L-glutamine, Trypsin-EDTA 0.25%, penicillin, and streptomycin were obtained from Gibco, Germany). All labwares for cell culture were procured from SPL Bioscience, Korea. Some regents for adipogenic and osteogenic differentiation, including ascorbic acid, β-glycerophosphate, dexamethasone, and fibroblast growth factor (FGF), were purchased from Sigma-Aldrich Company, Germany), while others, including knockout serum, Insulin-Transferrin-Selenium, and non-essential amino acid NEAA were prepared from Gibco. Phycoerythrin-labeled mouse anti-human CD105 and CD73, as well as FITC-labeled mouse anti-human CD34 and CD45 antibodies were bought from the eBioscience Corporation (USA).

### MSCs culture

hESC-MSCs at passage 2 were obtained from Royan Institute of Iran. The cells were grown in a low-glucose DMEM culture medium supplemented with 10% FBS, 2 mML-glutamine, 1,000 µ/ml penicillin, and 1,000 mg/ml streptomycin. The medium was changed every three days. ESC-MSCs were plated into cell culture flasks at a seeding density of 1 × 10^4^ cells/cm^2^. After seven days of growth, the confluent cultures were rinsed with Dulbecco PBS and incubated at 37 °C with Trypsin/EDTA solution (0.25%) for 3 min to detach the cells from the flask surface for subsequent passaging. hESC-MSCs in this study were of polyclonal origin.

### Flowcytometry

For immunophenotyping, 80% confluent fibroblast-like cells at passage 4-6 were washed with warm PBS and separated by Trypsin (0.25% with EDTA). After centrifuging, cell pellets were dissolved in PBS containing 2% FBS and incubated at 37 °C for 20 min. Then 2 μl of isotype control and antibodies (CD73, CD105, CD45, and CD34) was separately added to vials containing 2 × 10^5^ cells and kept at 4 °C for 30 min. The cells were washed with PBS and fluorescent-labeled cells were tested using FACS Calibur flow cytometer (Becton Dickinson, Germany) and analyzed by the Flowing software (ver. 2.4).

### Osteogenic differentiation of ESC-MSCs

For osteogenic differentiation, 3 × 10^3^ hESC-MSCs/cm^2^ were cultured in the osteogenic medium in 5% CO_2_ and 20% O_2_ at 37 °C for 21 days. Osteogenic medium consisted of DMEM was supplemented with 5% FBS, 200 μM ascorbic acid, 10 mM β-glycerophosphate, and 0.1 μM dexamethasone. After methanol fixation, the cells were stained with Alizarin Red and observed by an inverted microscope.

### Adipogenic differentiation of ESC-MSCs

For adipogenic differentiation, 1 × 10^4^ cells/cm^2^ were grown in DMEM/F12 medium containing 20% knockout serum, 2 mM L-glutamine, 1% NEAA, 0.1 mM β-mercaptoethanol, 100 ng/ml FGF, and 1% Insulin-Transferrin-Selenium in 5% CO_2_ and 5% O_2_ (hypoxia) at 37 °C for 14 days. After araformaldehyde fixation, the cells were stained with Oil Red O and assessed by an inverted microscope.

### hESC-MSC aggregation

To prepare aggregates, cells were non-adherently cultured for four days. ESC-MSCs (2.5 × 10^5^) suspended in 1 ml of culture medium were plated in agarose (0.1%)-coated plates. To assess cell numbers in different days, the aggregates were incubated with Trypsin/EDTA for 15 min while pipetting every 2-3 min, and dissociated cells were counted by a hemacytometer.

### Preparation of secretomes

Adherent or aggregated cultures were washed three times with Dulbecco PBS solution. Subsequently, 12 ml of DMEM supplemented with 0.05% human serum albumin and 2 mM L-glutamine was added to the cultures. The cultures were allowed to grow for 24 hours. After collecting CM and the removal of cell debris, the medium was concentrated to approximately 15× by centrifugation at 4000 ×g through a centrifugal ultra-filter unit with a cut-off of <3 KD (Millipore, USA). The concentrated CM was immediately cryo-preserved at -80 °C until use.

### Isolation and treatment of PBMCs (peripheral blood mononuclear cells)

PBMCs were isolated from 10 ml of freshly-isolated peripheral blood through Ficoll gradient (density = 1.077 g/cc; GE Healthcare, UK). PBMCs (1 × 10^5^) per well was seeded into 96-well plates with an equal volume of 50 μl from different secretomes and C10 culture medium (RPMI, 10% FBS, 1% L-glutamine, 1% NEAA, 100 U/mL penicillin, 100 μg/mL streptomycin, and 0.1 mM β-mercaptoethanol (Sigma). After incubating with the aggregated ESC-MSCs-derived secretome (aggregate-CM) and adherent ESC-MSCs-derived secretome (adherent-CM) for 18 hours, PBMCs were stimulated with 30 μg/ml of lipopolysaccharide (LPS) for 5 hours, and IL-1β, IL-10 and tumor necrosis factor α (TNF-α) secretion into the supernatant was measured via ELISA. LPS-induced PBMCs with no treatment were considered as a negative control group.

### ELISA

The ELISA kit (Thermo Scientific, USA) was used according to the manufacturers’ instructions. Production of IL-1β, IL-10, and TNF-α by PBMCs was determined by ELISA, and absorbance was measured at 450 nm using a microplate reader. Results were then compared with a standard curve plotted absorbance (Y-axis) against the concentration (X-axis).

### Statistical analysis

Data were analyzed by an unpaired student’s *t*-test (GraphPad Prism, version 6). Data were represented the mean of the samples ± standard deviation (SD). *p* values <0.05 were considered to be statistically significant.

## RESULTS

### ESC-MSC characterization

**Fig. 1:**
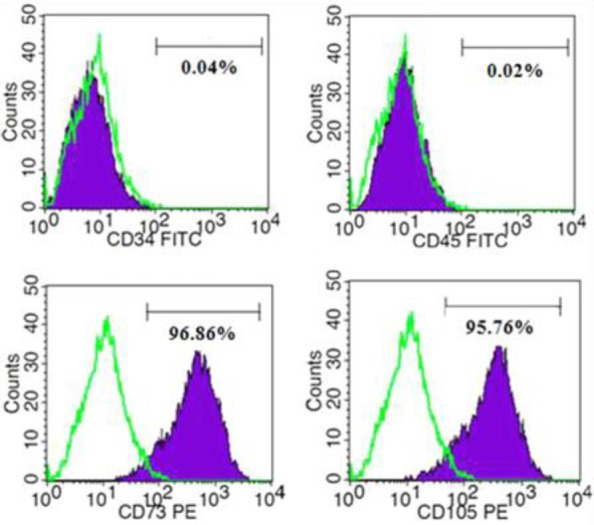
Analysis of surface antigens. Flow cytometry of ESC-MSCs was performed with phycoerythrin (PE)-conjugated antibodies for CD73 and CD105 and FITC-conjugated antibodies for CD34 and CD45 markers. The expression of isotype controls is shown as green histograms.

**Fig. 2:**
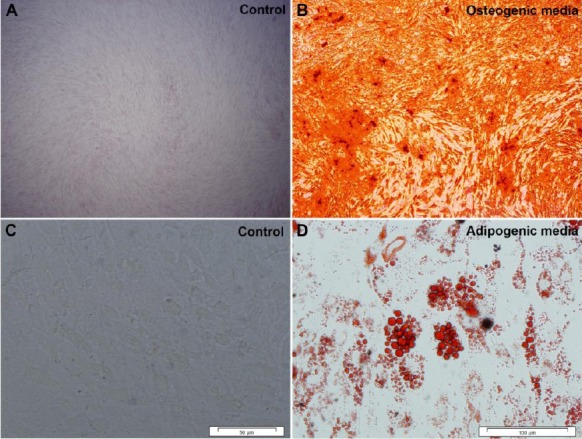
*In vitro* differentiation of hESC-MSCs. Osteogenic and adipogenic differentiation are presented by Alizarin red (A and B) and Oil Red O (C and D) staining, respectively. Cells cultured in non-adipogenic and -osteogenic media were considered as controls.

**Fig. 3:**
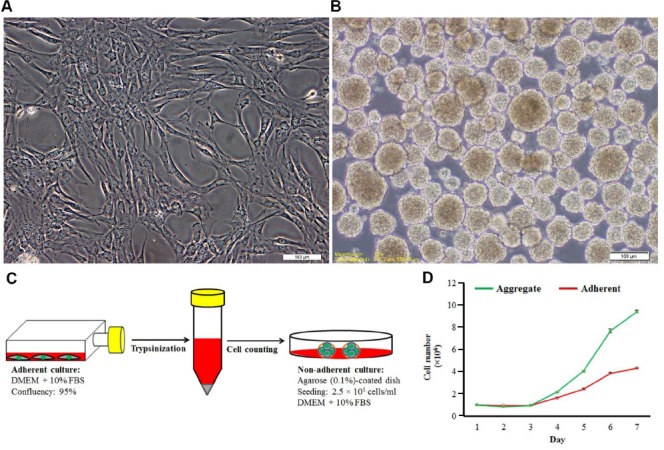
hESC-MSC aggregation. Cells in adherent (A) and non-adherent culture (B), scale bars = 100 um; (C) schematic representation of the non-adherent culture method; (D) the growth profile of hESC-MSCs in adherent (red) and non-adherent (green) cultures.

Flow cytometry was applied to evaluate cell surface markers; results showed that cells were negative for hematopoietic markers (CD34 and CD45) but positive for mesenchymal markers (CD73 and CD105) (Fig. 1). After culturing the cells in osteogenic and adipogenic media, it was found that MSCs were capable of differentiating into adipogenic and osteogenic lineages (Fig. 2). Additionally, non-adherently cultured ESC-MSCs showed the ability to form aggregation. To evaluate aggregate growth rates, the number of aggregates was counted using a hemocytometer on different days of culture. Our findings showed that the aggregate growth started approximately at the second day with increased numbers of aggregates (Fig. 3).

### Anti-inflammatory effect of hESC-MSC aggregate secretome

**Fig. 4:**
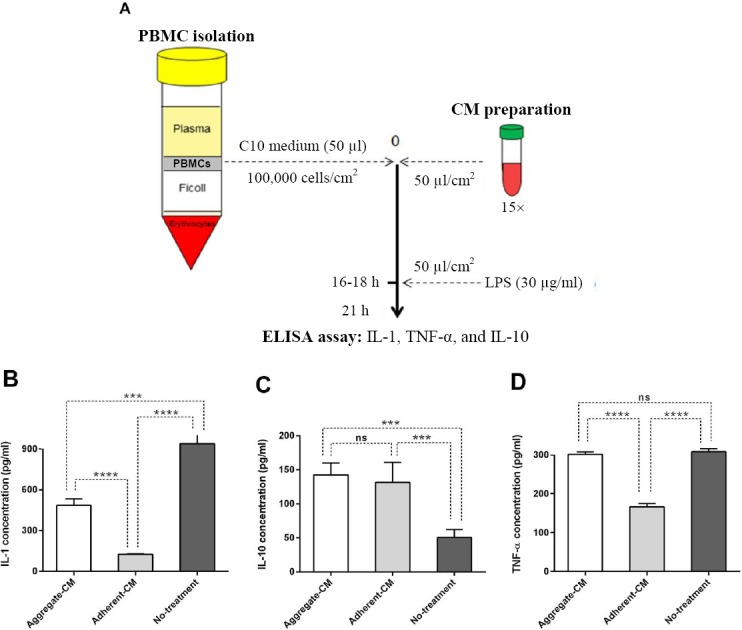
Effects of secretory molecules derived from hESC-MSC aggregates on PBMCs. (A) Schematic representation of the in vitro model to assess anti-inflammatory effects of hESC-MSCs. (B) ELISA assay to study IL-1β, (C) IL-10, and (D) TNF-α secretion from PBMCs after treatment with secretome prepared from non-adherent and adherent culture of hESC-MSC. PBMCs with no treatment were used as control group. Aggregate-CM, LPS-induced PBMCs treated with aggregated MSCs-derived secretome; adherent-CM, LPS-induced PBMCs treated with adherent MSCs-derived secretome; no treatment, untreated LPS-induced PBMCs; Bars are mean ± SD, one-way ANOVA with Turkey’s tests were used for multiple comparisons, n = 3, ^***^p < 0.001, ^****^p < 0.0001. ns, non-significant

To measure the concentration of IL-10, TNF-α, and IL-1β in the supernatant of PBMCs, ELISA was carried out following the treatment of PBMCs with aggregate-CM and adherent-CM. Results showed that PBMCs treated with aggregate-CM significantly decreased IL-1β (*p* < 0.0001) compared to the untreated group, but slightly decreased IL-1β secretion (*p* < 0.001) in comparison to the adherent-CM group (Fig. 4). In addition, PBMCs treated with aggregate-CM were able to secrete IL-10 as equal as adherent-CM, both showing a significant difference (*p* < 0.001) compared to the untreated group (Fig. 4). Surprisingly, PBMCs treated with adherent-CM exhibited a significant decrease in TNF-α secretion (*p* < 0.001) as compared to the untreated group; in contrast, PBMCs treated with aggregate-CM displayed no significant change compared to the untreated group (Fig. 4). Mean ± SD values of different groups are represented in Table 1.

**Table 1:** Mean ± SD values of no-treatment, adherent-CM, and aggregate-CM groups

Group	IL-1β (mean ± SD)	TNF-α (mean ± SD)	IL-10 (mean ± SD)
No-treatment	938.0 ± 131.0	307.9 ± 7.71	50.56 ± 11.62
Adherent-CM	125.2 ± 2.73	166.6 ± 8.04	131.6 ± 29.1
Aggregate-CM	485.2 ± 48.38	301.7 ± 5.906	142.5 ± 17.41

## DISCUSSION

The methods to increase the secretion of desired trophic and immunomodulatory factors and to enhance paracrine effects of MSCs include different preconditioning strategies such as physiological (e.g. hypoxia), molecular (treatment with cytokines and growth factors), pharmacological (e.g. lipopoly-saccharide), and physical preconditioning[19]. The 3D culture of MSC spheroids (aggregation) can also have a profound impact on the MSC secretome[21]. In this study, we presented evidence to support that the secreted molecules from hESC-MSC aggregates could modulate inflammation *in vitro*. The cells used in the present study were MSCs derived from hESCs; these cells displayed a fibroblastic morphology in 2D culture and expressed mesenchymal surface markers but failed to express hematopoietic markers. Furthermore, the hESC-MSCs were able to differentiate into multiple mesenchymal tissues *in vitro*. MSCs were originally isolated from bone marrow but are now known to be present in all fetal and adult tissues. However, tissue-isolated BM-MSCs require invasive procedures for harvesting marrow. Derivation of MSCs from hESCs has no invasiveness, and an unlimited amount of MSCs can be produced from hESCs. hESC-MSCs have been shown to possess characteristics similar to BM-MSCs. Recently, several studies have surveyed the immunomodulatory characteristics of hESC-MSCs. Yen et al.[22] found that hESC-MSCs could suppress lymphocyte proliferation and cytotoxic effects of activated NK cells, similar to BM-MSCs. In addition, Kimbrel et al.[23] showed that hESC-MSCs could modulate dendritic cells and could enhance regulatory T-cell populations. It has been observed that administration of MSC secretome could augment anti-inflammatory cytokines (e.g. IL-10) and decrease proinflammatory cytokine levels (e.g TNF-α and IL-1β) levels in sera of animal models[24,25]. On these discoveries, Milwid et al[26] and Jiao et al.[27] designed an in vitro assay in which PBMCs were first treated with BM-MSC secretome, followed by assessment of IL-10 secretion (as an immunomodulation indicator) from LPS-induced PBMCs by ELISA. This is a powerful tool to determine the immunomodulatory potency of MSCs in different preparations. Using such an in vitro assay, we have previously shown that the secretome of hESC-MSCs could have immunomodulatory potential[28]. In the present study, we applied the in vitro assay to determine whether aggregation preconditioning can affect immuno-modulatory characteristics of hESC-MSCs in comparison with 2D monolayer adherent culture. In addition to IL-10, we assessed secretion of proinflammatory cytokines such as TNF-α and IL-1β from PBMCs.

Our results indicated that aggregation of hESC-MSCs to spheroids could change secretome so that the immunomodulatory potency was significantly enhanced compared to secretome from 2D culture of hESC-MSCs. In consistence with our study, Ylöstalo *et al*.[29] has explored the effects of secretome extracted from BM-MSC spheroids on macrophages and showed that the secretome could inhibit LPS-stimulated macrophages from secreting pro-inflammatory cytokines such as TNF-α, CXCL2, IL-6, IL-12p40, and IL-23. They have also demonstrated that aggregate-prepared secretome is able to increase the secretion of anti-inflammatory cytokines such as IL-10 and IL-1RA from macrophages.

Aggregation of MSCs allows facilitated cell-cell contacts and interactions between the MSCs and the extracellular matrix as an *in vivo* manner. It has been proposed that the presence of hypoxia in the core of MSC aggregates could increase the secretion levels of some cytokines and growth factors[21]. It has also been demonstrated that hypoxia preconditioning of BM-MSCs significantly increases the secretion of several secretory molecules, including trophic factors (e.g. vascular endothelial growth factor, basic fibroblast growth factor, and placental growth factor[30], and immunomodulatory factors (e.g. TGF-β)[31], compared with normoxic condition that results in increased MSC therapeutic effects. Bartosh *et al*.[20] have prepared MSCs in the form of spheroids in order to maximally express TNF-α stimulated gene/protein6 and the anti-inflammatory protein; they demonstrated that the therapeutic effect of MSCs in animal models of myocardial infarcts is related to TNF-α stimulated gene/protein6. Moreover, Potapova *et al*.[32] organized MSCs into 3D aggregates to increase the secretion levels of paracrine factors. They observed high concentrations of IL-11, as well as cytokines such as vascular endothelial growth factor, basic fibroblast growth factor, and angiogenin in the CM from MSC spheroids, as compared with CM from MSC monolayers[32].

In conclusion, our data suggested that 3D aggregated MSCs may be more effective than MSCs from adherent 2D cultures for inflammatory conditions. It seems that the formation of 3D cellular aggregates leads to enhanced therapeutic capacity of MSCs by promoted secretion of proangiogenic and chemotaxic factors and anti-inflammatory cytokines.
